# Breaking barriers in Parkinson’s care: the multidisciplinary team approach

**DOI:** 10.1007/s00702-024-02843-6

**Published:** 2024-10-17

**Authors:** Zvezdan Pirtošek

**Affiliations:** Department of Neurology University Medical Centre, Faculty of Medicine, University of Ljubljana, Ljubljana, Slovenia

**Keywords:** Parkinson's disease, Multidisciplinary teams, Patient-centered management, Healthcare integration, Barriers to multidisciplinary approach

## Abstract

Parkinson’s disease is a complex neurodegenerative disorder presenting a range of motor and non-motor symptoms that greatly impact both patients and caregivers. The diverse needs arising from these symptoms make a multidisciplinary team (MDT) approach crucial for effective management. This article explores the role and benefits of MDTs in Parkinson’s care, highlighting how collaborative models improve clinical outcomes and quality of life. MDTs integrate neurologists, nurse specialists, therapists, and other professionals to deliver comprehensive, patient-centered care. The inclusion of patients and caregivers fosters shared decision-making, enhancing health outcomes. However, challenges like limited controlled trials, lack of comprehensive guidelines, and under-referral remain. Innovative models, such as telehealth and community-based care, offer promising solutions, especially in underserved regions. The article advocates for further research and standardized guidelines to optimize the MDT approach for Parkinson’s disease.

## Introduction

Parkinson’s disease (PD) is a progressive neurodegenerative disorder characterized by both motor and non-motor symptoms, including psychiatric and sleep disturbances, pain, sensory issues, autonomic dysfunction, and cognitive impairments. These symptoms contribute to a significant burden on both patients and caregivers. The complexity and variability of these symptoms, along with comorbidities and polypharmacy, necessitate a multidisciplinary team (MDT) approach to comprehensively address the diverse needs of patients and caregivers (Carne et al. 2005a, 2005b; Pretzer Aboff and Prettyman, 2015).

A multidisciplinary approach ensures stringent adherence to established standardized assessment and follow-up protocols, clinical guidelines, and state-of-the-art interventions. This approach shifts the focus of management to the personal goals of the patient and the well-being of the caregiver, thereby reducing the disconnect between the perspectives of physicians, patients, and caregivers on the symptoms (Bhidayasiri et al. 2020). The MDT philosophy supports and empowers patients to take charge of their health, which has been found to have positive effects on health outcomes (Greenwell et al. 2015). Ideally, patients and caregivers should be fully integrated within the multidisciplinary team.

There is a general consensus that multidisciplinary care is essential for treating Parkinson’s disease (Post et al. 2011; van der Eijk et al. 2015; van der Marck and Bloem 2014; Qamar et al. 2017; Lidstone et al. 2020; Pirtošek et al. 2020). Furthermore, several recommendations, guidelines, and considerations for the organization of multidisciplinary clinical care teams for persons with Parkinson’s disease and their care partners have been developed, which can be tailored to specific environments, whether in a tertiary centre, hospital, or local community (Radder et al. 2020a; Aye et al. 2020).

The American Academy of Neurology (AAN) introduced a PD Quality Measurement Set, available online, which recommends that all persons with Parkinson’s disease should have access to a broad range of medical and allied health professionals for personalized, integrative, and multidisciplinary care (Chou et al., 2021). Emerging guidelines and quality standards support the incorporation of occupational therapy (Sturkenboom et al. 2014), physiotherapy (Domingos et al. 2018), and speech and language pathology (SLP) therapy and dysphagia care in Parkinson’s disease management (Schindler et al. 2021). Despite the limited number of studies, it is becoming increasingly clear that people with Parkinson’s disease benefit from such an approach, and it is therefore increasingly recommended in PD treatment guidelines (National Collaborating Centre for Chronic Conditions (UK), 2006).

## Purpose and scope of the article

This article explores the structure and impact of multidisciplinary teams (MDTs) in Parkinson’s disease care, highlighting their role in improving patient-centered management of both motor and non-motor symptoms. It examines the challenges of implementing MDTs, such as access issues and lack of standardization, while addressing innovative care models like telehealth and community programs. The goal is to advocate for more structured MDT guidelines to enhance comprehensive care for Parkinson’s patients across different settings.

## Methods

A systematic literature review was conducted to explore the role and impact of multidisciplinary team (MDT) care in Parkinson’s disease management. The search was carried out using the PubMed database, covering publications from the period 2000 to 2024. The search algorithm used was: “Parkinson’s disease” AND (“multidisciplinary” OR “integrative” OR “collaborative”) AND (“guidelines” OR “treatment outcome”). This search focused on identifying clinical trials, reviews, randomized controlled trials (RCTs), meta-analyses, systematic reviews, and other clinical articles. Studies were included if they provided data on MDT structures, roles, effectiveness, or guidelines specific to Parkinson’s disease care. Studies were excluded if they did not specifically focus on Parkinson’s disease or lacked information on MDT practices. Additionally, the reference lists of key articles were reviewed to identify further relevant studies, ensuring a comprehensive assessment of the existing literature on MDT care in Parkinson’s disease. The initial search yielded a broad set of studies, which were then screened based on titles and abstracts to assess their relevance to the topic of multidisciplinary team (MDT) care in Parkinson’s disease.

## Key evidence points

### The concept of multidisciplinary approach

Studies provide different concepts of Multidisciplinary Team (MDT) approaches in the management of Parkinson’s disease, reflecting a spectrum of care models that address the disease’s complexity and local specificities. The initial concept of a team-based approach to Parkinson’s disease management was first articulated by Carlson-Davis in 1977, emphasizing the role of various healthcare professionals in occupational therapy (Giladi et al. 2014). This early model was expanded by Gibberd et al. in 1981 through a controlled trial that highlighted the contributions of physiotherapy and occupational therapy in the management of Parkinson’s disease.

The term ‘multidisciplinary care’ encompasses various levels of interaction and communication among healthcare professionals, patients, and caregivers, aiming for a comprehensive care approach. These levels range from minimal interaction, seen in parallel practice where professionals work independently, to highly integrative approaches where professionals collaborate extensively (Radder et al. 2020a; Boon et al., 2004):

- Parallel Practice: Healthcare professionals operate independently, providing care based on their specific expertise with minimal interaction among team members.
- Consultative Practice: Professionals maintain independent roles but consult with one another as needed, sharing advice without jointly planning patient care.
- Collaborative Practice: A more interactive model where professionals share information and plan care together, ensuring cohesive management of patient needs.
- Coordinated Practice: Care is more organized and integrated, with systems in place to align the efforts of different professionals, enhancing communication and cohesion in patient care.
- Multidisciplinary Practice: Each member addresses patient care from their professional perspective, working in concert but often without direct interaction, to provide comprehensive care.
- Interdisciplinary Practice: Regular interactive meetings are conducted for shared decision-making, allowing team members to develop and implement a unified care plan based on collaborative problem-solving.
- Integrative Practice: The most holistic model, focusing on blending various disciplinary perspectives to provide patient-centered care that adapts continuously based on feedback and outcomes.

Various models of multidisciplinary teams include inpatient facilities, outpatient and day clinics, community rehabilitation facilities, and synchronized multiprofessional groups in the community.

There is an increasing trend for the multidisciplinary team approach to be performed in the community setting, at the patient’s home, or in a regional community centre. Here, physiotherapists, occupational therapists, speech and language therapists, nutritionists, psychologists, social workers, and other professionals plan and administer interventions individually but communicate and share information electronically. This model of synchronized multiprofessional treatment in the community is particularly patient-friendly but requires the active involvement of the coordinator and a high level of motivation from all team members (Keus et al. 2012).

An excellent example of MDT being integrated into regional networks as a multifaceted integrative model is the Dutch ParkinsonNet (Bloem et al. 2017), supported by expert clinics within that region. New care models are arising, such as hubs combining medical services and science (Okun et al. 2018).

Understandably, MDT is much more of a challenge in underdeveloped low-income countries. An interesting model set up in Colombia, called the Saturday in Motion model, is an on-site activity with a community approach and a multidisciplinary philosophy, which has had a positive impact on patient and caregiver quality of life (Muñoz et al. 2020).

### Roles and responsibilities of Team members

Studies provide different concepts of MDT roles and responsibilities in managing Parkinson’s disease, underscoring the importance of specialized knowledge and collaboration.

The **neurologist**’s role is pivotal due to their specialized knowledge and expertise in diagnosing and managing the complex neurological aspects of Parkinson’s disease. They are primarily responsible for diagnosing PD, conducting thorough clinical assessments, ordering and interpreting diagnostic tests, and initiating pharmacological treatments to manage both motor and non-motor symptoms. Neurologists also play a crucial role in monitoring disease progression and adjusting treatment plans as needed. In later stages, their expertise extends to overseeing the implementation of advanced therapies such as deep brain stimulation (DBS) and infusion therapies (Pirtošek et al. 2020). Given their central role in diagnosis and treatment, neurologists are well-suited to take a coordinating role within the MDT. They educate patients and their families about the disease, treatment options, and symptom management strategies, which is critical for empowering patients, facilitating adherence to treatment plans, and enhancing overall quality of life.

Neurologists are involved in research and clinical trials aimed at discovering new treatments and improving existing therapies for PD. Their participation in research helps integrate the latest scientific advancements into clinical practice, ensuring that patients benefit from cutting-edge treatments and strategies. This dual role in clinical practice and research highlights the neurologist’s comprehensive involvement in Parkinson’s disease care and management.

Practice identified the significant role of **Parkinson’s Disease Nurse Specialists (PDNS)** within a multidisciplinary team, highlighting their importance in providing detailed education and managing complex medication regimens. PDNSs monitor adherence, adjust dosages, and educate patients on the effects and timing of medications. They perform regular assessments using standardized tools to monitor disease progression and treatment effectiveness. Trained to address both motor and non-motor symptoms, PDNSs ensure comprehensive care and coordination among various specialists such as neurologists, physiotherapists, occupational therapists, and speech therapists. It has been noted that patients might feel more comfortable discussing sensitive issues, such as sexual dysfunction, with their PDNS than with their physician (Calne et al., 1994).

Different models showed that PDNSs play a critical role in introducing and managing advanced therapies such as infusion therapies and DBS. They provide both preoperative and postoperative care, manage complications, and support the use of telehealth services to reach patients in remote areas. This ensures that care plans are cohesive and tailored to the individual needs of each patient. The role of PDNSs is well-defined and integrated into patient care and education throughout Europe (e.g., Denmark, Netherlands, Slovenia, Nordic countries) and beyond (e.g., Australia, US, Thailand). Their roles are emerging even in low-income countries, such as Sub-Saharan Africa (Williams et al. 2018) and Sri Lanka (Persson 2024). The nEUROcare initiative, a European project for capacity building, aims to educate nurses for care of people with neurodegenerative disorders in Sri Lanka (Persson 2024). The UK provides the most structured role for PDNSs, with official recognition and structured training, allowing them to act as consultants with full prescribing authority and run specialized treatment clinics, reducing the workload on neurologists. PDNS operate in various settings, from tertiary centers to community care, adapting their roles to support patients at home. Their involvement in using the latest clinical technology has been reported to improve adherence to advanced therapies and maintain therapy efficacy (Chaudhuri and Fung 2016).

The role of PDNSs remains important across all stages of Parkinson’s disease (Ocepek 2002). In the early stages, they help explain the disease, its impact, and treatment options, including pharmacological testing for drugs like levodopa or apomorphine. As the disease progresses, their role becomes even more pivotal due to the increased demands of nursing and the need for coordination in device-aided treatments. These advanced stages require training, interaction, and coordination with patients, caregivers, and other MDT members. This stage also involves initiating discussions about advance care planning and palliative care.

With the emergence of more sophisticated device-aided therapies, PDNSs are increasingly specializing in areas such as DBS, apomorphine, or levodopa infusion therapy. These specialized roles, such as ‘DBS nurse,’ ‘Apomorphine nurse,’ or ‘Duodopa/Lecigon nurse,’ allow PDNSs to manage and coordinate these services effectively (Martin and Mills 2013; Bhidayasiri, 2016). However, PDNSs must have a broad understanding of various treatment needs beyond subspecialized device-aided interventions (Ocepek 2002).

Despite their significant roles, evidence from randomized controlled trials (Reynolds et al. 2000; Jahanshahi, 1994; Jarman et al. 2002; Connor et al. 2019) has so far been inconclusive about the direct impact of PDNSs on clinical outcomes. Nonetheless, the perception of their importance remains strong, and patients value their contribution (Connor et al. 2020).

On the other hand, the scientific evidence supporting **physical therapy** is abundant and persuasive. Benefit for physiotherapy was found in most outcomes over the short term in a Cochrane review (Tomlinson et al. 2013). A recent meta-analysis (Radder et al. 2020b) showed that conventional physiotherapy significantly improved motor symptoms, gait, and quality of life. Various forms of physical activity, including resistance training, treadmill training, dance, Nordic walking, balance training, and martial arts, have demonstrated effectiveness in improving motor symptoms, balance, and gait. Exergaming and hydrotherapy have also shown positive impacts on balance and quality of life. Ernst et al. (2024) provided evidence of beneficial effects on motor symptoms and quality of life from most types of physical exercise, although there was little evidence of differences between specific interventions. Specialized physiotherapy has been associated with fewer PD-related complications and reduced healthcare costs (Ypinga et al. 2018).

High-intensity aerobic exercise is particularly effective and is hypothesized to impact disease progression positively (Gamborg et al. 2022). Physical (and occupational) therapy plays a crucial role in providing compensation strategies, including external and internal cueing, altering mental states, action observation/motor imagery, adopting new walking patterns, and dual-task training (Nonnekes et al. 2019; Heinzel et al. 2016). Physical activity has also been shown to benefit non-motor symptoms like depression and sleep disturbances (Amara et al. 2020).

Technological advances, accelerated by the COVID-19 pandemic (Feeney al. 2021), have led to a shift toward telerehabilitation in Parkinson’s disease. Innovations such as virtual reality, wearable sensors, and machine learning-based signal processing are being integrated into patient care (Nuara et al. 2022).

**Occupational therapists** are integral in helping Parkinson’s disease patients maintain independence in activities of daily living, such as dressing, bathing, and eating (Sturkenboom et al. 2014). They assess home and work environments to recommend modifications that improve safety and accessibility. Tailoring the environment to the functional needs of the patient can prevent falls and other accidents, which are common in Parkinson’s disease due to motor difficulties and postural instability (Jonasson et al. 2015). Occupational therapists provide adaptive strategies to overcome challenges posed by motor symptoms like tremors and rigidity.

Research has shown that multidisciplinary intensive rehabilitation treatment significantly improves daily living activities and sleep performance in PD patients (Doucet et al. 2021). Other studies have confirmed the beneficial effects of upper limb therapy on short-term function (Welsby et al. 2019). Occupational therapy has demonstrated improvements in medication management, handwriting, participation in daily activities, sleep quality, and overall quality of life (Foster et al. 2021; Tofani et al. 2020). Clinical recommendations support multidisciplinary, goal-oriented interventions that include various forms of exercise, mindfulness meditation, and task-oriented training, integrating self-management and other cognitive-behavioral approaches (Wood et al. 2022).

Despite its effectiveness, occupational therapy remains underutilized in managing Parkinson’s disease. A study of over 5,000 patients in the United States and Canada found low referral rates to occupational therapy across all stages of Parkinson’s disease (Roberts et al. 2021). A SWOT analysis suggests that underutilization may be due to a lack of specific training, infrequent patient interactions, overlapping treatment objectives with other healthcare professions, and unfamiliarity with guidelines (Cavaglion et al. 2022).

**Speech therapists** (SLPs) play a critical role in managing speech and swallowing difficulties associated with Parkinson’s disease (Herd et al. 2012). Common issues include hypokinetic dysarthria and dysphagia, the latter having a high prevalence and leading to complications such as aspiration pneumonia (Takizawa et al. 2016). SLPs are skilled in addressing these problems early, using specific exercises and techniques, including the Lee Silverman Voice Treatment, which has been shown to improve vocal loudness and functional communication (Pu et al. 2021). Their role often overlaps with psychologists, as cognitive changes in Parkinson’s disease affect communication abilities.

A multidisciplinary consensus panel has provided guidance on dysphagia treatment in Parkinson’s disease, emphasizing collaboration among neurologists, otorhinolaryngologists, gastroenterologists, phoniatricians, SLPs, dieticians, and clinical nutritionists (Schindler et al. 2021). SLPs increasingly utilize telerehabilitation methods for managing speech and swallowing issues, demonstrating noninferiority to traditional, in-person treatment methods (Constantinescu et al. 2011; Theodoros 2021). This approach has been particularly valuable during the COVID-19 pandemic, highlighting the potential for effective remote therapy options.

Literature shows that non-motor symptoms significantly contribute to the diminished quality of life in Parkinson’s disease patients, with cognitive impairments and psychiatric conditions such as depression, anxiety, and psychosis being highly prevalent (Schrag et al. 2000). **Psychiatrists and psychologists** are crucial in managing these symptoms, with major depression affecting 17% of patients and anxiety disorders affecting 31–43% (Reijnders et al. 2008; Broen et al. 2016). These conditions can arise as reactions to diagnosis, intrinsic parts of Parkinson’s disease pathology, or side effects of pharmacological treatments.

Psychologists contribute by conducting cognitive assessments and providing interventions like cognitive-behavioral therapy (CBT), which has proven effective in improving mood and overall function (Troeung et al. 2014). They offer critical support and counseling to caregivers, helping them manage stress and caregiving responsibilities effectively (Rodriguez-Violante et al., 2015). In multidisciplinary settings, psychologists’ skills can also enhance team dynamics, improving the overall effectiveness of the team’s approach to managing Parkinson’s disease.

Psychiatric expertise is essential for managing severe mood disorders, impulse control disorders, psychosis, and dementia associated with Parkinson’s disease (Taylor et al. 2016; Aarsland et al. 2007). Psychiatrists are responsible for psychiatric diagnoses, prescribing psychotropic medications, and collaborating closely with neurologists to adjust antiparkinsonian medications, which can induce psychiatric symptoms. Effective management of these complex issues requires close interdisciplinary collaboration.

**Dieticians and nutritionists** provide valuable support within MDT care by offering personalized nutritional interventions, usually as a complement to primary interventions, as diet can influence both the onset and progression of the disease (van der Berg, 2024). Dietary factors impact the preclinical phase of Parkinson’s disease and have direct implications for managing motor symptoms and medication effectiveness. For example, protein intake can affect levodopa absorption, and diet can influence gastric emptying rates, contributing to motor and non-motor fluctuations. Nutritionists design individualized dietary plans to address issues like unintentional weight changes, bone health, constipation, and hydration, which are common in Parkinson’s patients (Barichella et al. 2009). Low-protein and protein-redistribution diets are particularly important for managing motor fluctuations and enhancing treatment outcomes (Cereda et al., 2010).

Emerging research suggests that dietary modifications may also have disease-modifying effects by influencing mitochondrial function, inflammation, immune responses, and the gut microbiome (Kleine Badenhorst et al., 2023). There is a growing interest in understanding the links between diet, pesticide exposure through the food chain, and Parkinson’s disease risk (Kab et al. 2017; Kulcsarova, 2023). However, more research is needed to establish definitive dietary guidelines for Parkinson’s disease management (van der Berg, 2024).

An efficient **coordinator** is key to the success of a multidisciplinary team, ensuring seamless integration and communication among team members. The coordinator, often a specialized PD nurse or neurologist, serves as the main point of contact, linking patients with the healthcare system across primary, secondary, and tertiary care settings. Effective coordinators possess leadership, communication, and organizational skills, enabling them to manage the complex needs of Parkinson’s disease patients and their families. Their role is vital in maintaining the coherence of care plans, aligning interventions across different disciplines, and ensuring that patient care is both comprehensive and individualized.

### Benefits of multidisciplinary approach

Studies provide different concepts of multidisciplinary care, illustrating its substantial benefits in managing Parkinson’s disease (PD). Evidence suggests that multidisciplinary care improves symptom management, enhances quality of life, prevents complications, and provides valuable education and support to both patients and caregivers.

Practice identified significant improvements in symptom management through team-based care in PD, affecting various outcomes such as functional status, fall rates, self-efficacy, and both motor and non-motor symptoms (Krause et al. 2022). Additionally, these benefits extend to enhancing caregiver well-being (Lidstone et al. 2020). Carne et al. (2005a) found that nearly three-quarters of PD patients experienced maintenance or improvement in motor function within the first year of multidisciplinary care, supplementing the benefits of standard PD medication. Furthermore, Carne et al. (2005b) reported that these improvements could be sustained for up to three years after beginning a multidisciplinary program. White et al. (2009) highlighted that interdisciplinary rehabilitation positively impacted walking activity and endurance, depending on patients’ initial walking levels. Moreover, Ellis et al. (2008) demonstrated the efficacy of inpatient multidisciplinary rehabilitation programs specifically designed for people with Parkinson’s disease.

Different models showed that multidisciplinary care enhances the quality of life for PD patients, confirmed by various studies over the years. Early research (Trend et al. 2002; Wade et al. 2003; Carne et al. 2005b; Tickle-Degnen et al. 2010) indicated positive effects of short-term multidisciplinary interventions on quality of life and behavioral outcomes. Recent studies have reaffirmed these findings, demonstrating sustained quality of life improvements through multidisciplinary care models (van der Marck et al. 2013a; Marck et al. 2013b; Krause, 2022). The American Academy of Neurology supports this view, suggesting that collaborative care models significantly enhance both quality of life and functional outcomes in PD patients (Chou et al. 2021). Additionally, improved quality of life has been documented for caregivers involved in multidisciplinary care settings (Martinez-Martin, 2012; Wade et al. 2003; White et al. 2009).

### Guidelines

Various guidelines emphasize the critical role of multidisciplinary team (MDT) involvement in Parkinson’s disease care​ (https://www.nice.org.uk/guidance/ng71). However, these guidelines often focus on specific aspects, such as the roles of individual MDT members, specific interventions, or quality of care delivery, rather than providing a comprehensive, holistic framework that encompasses the entire spectrum of MDT collaboration, care coordination, patient-centered approaches, cost-effectivness and dynamic team functioning. Published standards exist for individual disciplines, including occupational therapy (Sturkenboom et al. 2014), physiotherapy (Domingos et al. 2018), and speech and language pathology (SLP), including dysphagia care (Schindler et al. 2021), each addressing their unique contributions within the MDT. The National Collaborating Centre for Chronic Conditions (UK) (2006) recommends a multidisciplinary approach to Parkinson’s disease management, underscoring the benefits of a team-based, holistic care model that not only treats symptoms but also supports patients and caregivers through comprehensive, coordinated strategies. Similarly, the American Academy of Neurology (AAN) developed a Quality Measurement Set for improving care and outcomes in Parkinson’s disease (Factor et al. 2016). This set highlights specific roles and practices for MDT members and identifies areas for improvement, yet stops short of offering an overarching guideline that integrates all facets of MDT care—such as the continuous evolution of care plans, fluid communication across specialties, and alignment of individual expertise towards unified goals.

In exploring the complexities of team-based care, van der Marck et al. (2014) discuss essential components like team composition, collaboration forms, interdisciplinary communication, and the practical delivery of multispecialty care. However, the article underscores the inherent challenges of developing such a multifaceted model within the constraints of modern healthcare systems, which can be as complex as the disease itself.

More recently, twenty expert centers specializing in multidisciplinary Parkinson’s care produced 30 key recommendations, emphasizing the need for patient-centered care, the availability of a core MDT for every newly diagnosed patient, and the appointment of a dedicated team coordinator to ensure the fluidity and continuity of care (Radder et al. 2020a).

### The caregiver’s burden

Holistic management of Parkinson’s disease patients, incorporating the role of caregivers, remains a key aspect of multidisciplinary care. Although caregivers contribute significantly to the effectiveness of the multidisciplinary team, studies have reported mixed results concerning caregiver strain. While some studies found no significant effect (Trend et al. 2002; Wade et al. 2003), others observed a negative impact (van der Marck, 2013a, 2013b). This negative perception could stem from the increased awareness of PD’s complexity and the demand to manage multiple appointments and participate actively in various interventions, as observed by Tosserams et al. (2020). Despite these challenges, caregiver involvement is essential for comprehensive care, given their critical role in the daily support and management of PD patients.

### Multidisciplinary team approach: case study of Ljubljana

To illustrate the implementation of these multidisciplinary care principles, we examine the case of the Ljubljana University Medical Centre MDT, which has successfully adopted this approach for over three decades.

The Ljubljana University Medical Centre MDT was established in 1988 as part of an emerging tertiary Movement Disorders Centre at the University Hospital. Initially, the team consisted of one neurologist, one Parkinson’s disease nurse specialist (PDNS) (Ocepek 2000), one physiotherapist, and one occupational therapist. In 1988, this MDT was the initiator of the foundation of the Slovenian Association of Patients with Parkinsonism Trepetlika (engl. *Trembling poplar*). Today, MDT includes core members such as six movement disorder neurologists, five PDNSs, two occupational therapists, three physiotherapists, a psychiatrist, a (neuro)psychologist, a social worker, and two speech and language therapists. Apart from the five PDNSs with expert knowledge in all movement disorders and interventions, there are two nurses dedicated to deep brain stimulation (DBS) intervention, known as ‘DBS nurses’. The education of PD nurses is not nationally formalized, but it is well organized and partially regulated through the Centre for Extrapyramidal Disorders.

Additionally, allied members are invited as needed, including a gastroenterologist, neurosurgeon, neurologist, dermatologist, dietitian, neuro-urophysiologist and pharmacologist. The senior neurologist serves as the team leader, and the senior PDNS, with experience in PD and good communication and organizational skills, acts as the coordinator. The coordinator is readily available to patients, their families, and other team members. The PDNS role is well-defined and known to all team members, patients, and caregivers (Ocepek 2002).

The team benefits from its decades-long tradition of the MDT approach, which has reinforced good understanding, respect for each other’s roles, and mutual trust among team members. The MDT convenes in regular face-to-face meetings every week to discuss complex patients and other issues such as guidelines, new treatments, and organizational matters. Any team member can bring up inpatient or outpatient issues for team discussion.

These formal meetings are crucial for coordinated care and strategic planning. However, a significant portion of the work, consultation, and information sharing occurs informally during coffee breaks and other daily activities, in a more relaxed setting. This spontaneous exchange of ideas and experiences fosters adaptability within the team, leading to innovative solutions and ultimately improving patient care.

An important part of the Ljubljana team is a specific PD nurse consultancy. Patients are seen by the neurologist at least once a year, but they may see the PDNS in her consultancy, as often as needed, and PDNS can refer them to other team members. The multidisciplinary team provides specialized services in all clinical, educational, and research aspects of PD management and closely cooperates with Trepetlika, the national patients’ organization.

Twice per year, the coordinator PDNS organizes a three-day PD Seaside Camp for patients, caregivers, and team members. This is an excellent opportunity for team members to interact with patients in an informal environment, along with their spouses or friends. During the camp, team members deliver lectures on various topics related to the symptomatology of parkinsonism, and patients participate in sessions such as physiotherapy, Tai Chi, dance, table tennis, musical therapy, healthy cooking courses, and similar activities. These activities aim to enhance patients’ daily activities and improve their quality of life, as well as that of their caregivers. Some of the camps are thematic, focusing on specific groups such as newly diagnosed patients, patients with advanced PD, caregivers, or patients with young-onset PD. Activities and meetings are then tailored to the particular needs and issues of these groups.

## Discussion: challenges and barriers to implementing multidisciplinary care

Despite anecdotal support and clinical intuition pointing to the effectiveness of MDTs, there are substantial challenges and barriers that need to be systematically addressed:

### Lack of controlled trials and clear evidence

One of the foremost challenges is the scarcity of well-designed controlled trials that rigorously evaluate the benefits of multidisciplinary care for PD. The existing studies exhibit considerable heterogeneity in terms of research design, intervention methods, and outcome measures. For instance, while some studies indicate substantial improvements in quality of life and motor function (Eggers et al. 2018; Ferazzoli et al.,^2018^), others fail to demonstrate similar benefits (van der Marck, 2013b). This variability complicates the task of synthesizing data and drawing definitive conclusions about MDT efficacy (Hagell 2007).

To bridge this gap, future research must focus on conducting more rigorous trials with standardized protocols and outcome measures. Larger sample sizes and longer follow-up periods will also be necessary to provide robust evidence regarding the long-term impact of multidisciplinary care on PD patients. Such methodologically sound research is critical to substantiating the benefits of MDTs in PD management.

### Adapting organizational models for multidisciplinary care

Traditional MDTs are predominantly based in specialized tertiary care centers, which may not be suitable for all patient populations. Alternative models, such as inpatient rehabilitation (Monticone et al. 2015; Ferrazzoli et al. 2018), community-based care (van der Marck et al. 2013b; Munnecke et al., 2010), home visits (Fleisher et al. 2018; Dorsey et al. 2016), hubs combining medical services and science (Okun et al. 2018) need to be explored and adapted to make PD care more patient-centered. These models can help alleviate the logistical and emotional burdens on patients and caregivers, especially those living in remote or underserved areas.

Home-based care, facilitated by in-person visits or telehealth solutions, is gaining traction as a more patient-friendly approach (van den Bergh et al. 2021). The COVID-19 pandemic (Ross et al. 2022) has accelerated the adoption of telehealth technologies and innovative virtual platforms (Ferestehnejad et al., 2022; Schneider et al. 2020), demonstrating their potential to enhance patient care while reducing the need for frequent hospital visits (Miner et al. 2021; Zhang et al. 2021). However, the effective implementation of such models requires training healthcare professionals in the use of telehealth tools and overcoming technological barriers. While telehealth and virtual systems can enhance access and efficiency in MDT care, it is important to consider the cognitive status of Parkinson’s patients. Those with significant cognitive decline or dementia may find it challenging to engage with telemedicine or digital platforms, potentially limiting the feasibility and effectiveness of these approaches. These patients may require more traditional, in-person approaches or additional caregiver support to engage effectively in MDT care.

### Complexity of multidisciplinary care models

The MDT approach is very time-consuming and full implementation of an MDT approach may not be feasible in all healthcare systems. Implementing effective MDT care requires navigating complex organizational structures, involving highly qualified professionals with specialized knowledge in PD. To improve feasibility and address resource and time constraints in MDT implementation, a tiered model could be utilized. This model allows for a basic core MDT in resource-limited settings, with more comprehensive care available where resources allow. Strategies to facilitate effective communication among MDT members, such as telehealth services and streamlined communication processes, can facilitate MDT collaboration without adding undue burden.

As the high cost of specialized care models may not always translate into better outcomes, prompting the need for innovative solutions to optimize cost-effectiveness and care delivery (Okun et al. 2018). Models like the interdisciplinary service and science hub at the University of Florida offer promising frameworks by integrating communication, preventive care, outcome tracking, and patient care optimization into a cohesive system.

### Under-referral and late referral to MDTs

Another challenge is the underutilization of MDTs, with many patients being referred too late, typically only after significant complications arise (Roberts et al. 2021). This under-referral results in missed opportunities for early, proactive interventions that could enhance patient outcomes. Educating healthcare providers on the benefits of early, multidiscipline referrals and developing standardized referral pathways are essential to improve patient access to comprehensive care.

### Educational gaps and need for training

There is a notable lack of specific educational programs focusing on MDT care for both healthcare providers and patients with PD. These gaps hinder the ability to deliver optimal care and engage patients and caregivers effectively in the care process (Cavaglion et al. 2022). Educational initiatives, such as the Swedish National Parkinson School, which emphasize patient and caregiver education, can significantly enhance the effectiveness of MDT care by empowering patients and their families to actively participate in healthcare decisions (Hellqvist et al., 2018).

While the involvement of family members is crucial, it is recognized that this may vary depending on cultural and socioeconomic contexts. Practical support, such as educational resources and connection to support services, is encouraged for families with limited availability.

### Maintaining positive Group dynamics

For MDTs to function effectively, fostering a collaborative environment with mutual respect, trust, and understanding among team members is crucial (Radder et al. 2020a). Overcoming traditional hierarchical structures in medical settings can promote a culture of equality, inclusivity, and open communication. Regular team-building activities and structured opportunities for interdisciplinary collaboration can further enhance team dynamics and improve patient care.

### Evaluating cost-benefit and emerging challenges

The financial implications of MDTs remain a concern, with studies showing inconclusive results regarding cost-effectiveness. However, considering indirect savings such as reduced emergency visits, hospital admissions, and better overall disease management is essential. Moreover, emerging challenges, such as integrating telemedicine and other technological advances, require MDTs to adapt and innovate continually (Schneider et al., 2017; Xu et al., 2021).

### Addressing caregiver burden and supporting underserved populations

Caregiver burden is a significant concern in PD management, often leading to stress and burnout. Providing better support systems and educational resources for caregivers is crucial for sustainable MDT care. Additionally, underserved populations face unique challenges in accessing MDT services. Implementing community-based care models and leveraging telemedicine can help extend MDT services to these populations, ensuring equitable access to high-quality care.

### Need for comprehensive guidelines

The absence of universally accepted guidelines for multidisciplinary care in Parkinson’s disease is another significant barrier. Various guidelines recommend that all persons with PD should have access to a broad range of medical and allied health professionals and several different organizational models of multidisciplinary care have been evaluated. Variations in current care approaches contribute to inconsistencies in treatment quality and effectiveness. To address these disparities, comprehensive guidelines should focus on the following key strategies: (i) *standardization across models*: align diverse MDT models (e.g., inpatient, community, telehealth) to ensure consistent care quality across all settings; (ii) *clear MDT roles*: define specific responsibilities for all MDT members, facilitating coordinated care and preventing overlap; (iii) *early referral pathways*: highlight protocols for early MDT referral to improve timely and effective care, reducing under-referral; (iv) *adaptability to resources*: ensure guidelines are adaptable to different healthcare settings, including low-income and underserved areas; (v) *integration of telehealth*: recommend effective use of telehealth and emerging technologies to enhance MDT care access and efficiency; (vi) *education and training*: address educational gaps with training for professionals on teamwork and interventions, plus patient/caregiver education to promote active care involvement; (vii) *research and evidence-based practice*: encourage rigorous research and uniform outcome measures to provide strong evidence for MDT benefits in PD care; (viii) *cost-effectiveness*: focus on cost-benefit optimization, considering indirect savings and innovative models to sustain MDT care; (ix) *caregiver support*: strategies to manage caregiver burden with resources and programs for better involvement and reduced stress; (x) *regular MDT review*: promote frequent team meetings for ongoing review, ensuring care plans stay patient-centered and adaptable.

## Conclusion

Parkinson’s disease requires a multifaceted care approach, and the strengths of multidisciplinary teams (MDTs) are increasingly evident. MDTs provide holistic, patient-centered care that addresses motor and non-motor symptoms, improves quality of life, and enhances adherence to clinical guidelines. While current barriers, including limited controlled trials, varied guidelines, and under-referral, exist, these challenges underscore the need for standardized protocols and further research to substantiate MDT benefits. Additionally, adapting models such as telehealth and community-based approaches can enhance care accessibility and cost-effectiveness. By addressing these areas and committing to continuous improvement, MDTs have the potential to significantly improve care and quality of life for Parkinson’s disease patients and their caregivers.

## Data Availability

Data sharing not applicable.
